# Population Genomics and Haplotype Analysis in Bread Wheat Identify a Gene Regulating Glume Pubescence

**DOI:** 10.3389/fpls.2022.897772

**Published:** 2022-07-13

**Authors:** Xin Hu, Jianfang Zuo

**Affiliations:** ^1^The Key Laboratory for Quality Improvement of Agricultural Products of Zhejiang Province, College of Advanced Agricultural Sciences, Zhejiang A&F University, Hangzhou, China; ^2^College of Plant Science and Technology, Huazhong Agricultural University, Wuhan, China

**Keywords:** wheat, glume hairiness, *Hg1*, GWAS, haplotype

## Abstract

Glume hairiness or pubescence is an important morphological trait with high heritability to distinguish/characterize wheat and is related to the resistance to biotic and abiotic stresses. *Hg1* (formerly named *Hg*) on chromosome arm 1AS controlled glume hairiness in wheat. Its genetic analysis and mapping have been widely studied, yet more useful and accurate information for fine mapping of *Hg1* and identification of its candidate gene is lacking. The cloning of this gene has not yet been reported for the large complex wheat genome. Here, we performed a GWAS between SNP markers and glume pubescence (Gp) in a wheat population with 352 lines and further demonstrated the gene expression and haplotype analysis approach for isolating the *Hg1* gene. One gene, *TraesCSU02G143200* (*TaELD1-1A*), encoding glycosyltransferase-like ELD1/KOBITO 1, was identified as the most promising candidate gene of *Hg1*. The gene annotation, expression pattern, function SNP variation, haplotype analysis, and co-expression analysis in floral organ (spike) development indicated that it is likely to be involved in the regulation of glume pubescence. Our study demonstrates the importance of high-quality reference genomes and annotation information, as well as bioinformatics analysis, for gene cloning in wheat.

## Introduction

Bread wheat (*Triticum aestivum*, 2*n* = 6*x* = 42, AABBDD) is an important cereal crop and is used as a staple food all over the world. It originated from two independent hybridization and polyploidization events. The first hybridization between wild einkorn (*Triticum urartu*, AA genome) and a close relative of *Aegilops speltoides* (SS≈BB genome) formed the tetraploid wild emmer (*Triticum dicoccoides*, AABB genomes), and the second hybridization happened between domesticated emmer (*Triticum dicoccum*, AABB genomes) and the wild goat grass (*Aegilops tauschii*, DD genome), which formed hexaploid bread wheat (Morris and Sears, 1967; Gill and Friebe, 2002; Matsuoka, 2011). During the evolution and domestication of wheat, many key morphology traits (such as brittle rachis, tough glume, and free-threshing) controlled by single major genes (*Br/br*, *Tg/tg*, and *Q/q*) (Gill et al., 2007) were firstly domesticated to meet the agricultural activities; then, additional quantitatively inherited traits, e.g., grain yield, seed size, plant height, and heading date meeting the human needs, were modified during domestication and the subsequent breeding process.

Hairy glume, also known as pubescent glume, appears in diploid, tetraploid, and hexaploid species in the *Triticeae* tribe (Tsunewaki, 1966). Hairy glume can be used as a phenotypic trait (or marker) for the evaluation of distinctness, uniformity, and stability of wheat cultivars (*T. aestivum* L.) due to its characteristics such as easy observation and independence of environmental effects (Parker and Namuth-Covert, 2017). It used to be scored as a trait to study the phenotypic diversity of tetraploid (Eticha et al., 2005; Hailu et al., 2006; Mengistu et al., 2015) and hexaploid wheat (Zeven and Schachl, 1989; Geleta and Grausgruber, 2011). Moreover, hairy glume has shown linkage to important genes/loci such as barley yellow dwarf virus (BYDV) resistant gene (Wu et al., 1999), powdery mildew resistance gene (*Pm3*) (Briggle and Sears, 1966), leaf rust (Howes, 1986) and Karnal bunt (Warham, 1988), tiller inhibition gene (*Tin*) (Richards, 1988; Spielmeyer and Richards, 2004), *Gli-A1* locus (Howes, 1986), and abiotic stress gene loci (like cold and drought) (Trethowan et al., 1998; Pshenichnikova et al., 2019); therefore, it was frequently used as a morphological marker to assist mapping of these genes/loci. Several pieces of research indicated that the ratio of hairy glume in tetraploid wheat is greater than that in hexaploid wheat (Tsunewaki, 1966; Jain et al., 1975; Zeven, 1990; Ruiz et al., 2002; Hailu et al., 2010), which implied that hairy glume has been under selection for a certain evolutionary extent.

Glume hairiness or pubescence is an important morphological trait with high heritability to distinguish/characterize wheat, and its genetic analysis could date back to the early decades of the 20th century. The hybrid experiment between the felted glume (hairy glume) and glabrous glume wheat performed by Biffen (1905) reported that felted glume was dominant over glabrous glume. The separation ratio of hairiness: glabrous was 3:1 in an F_2_ population experiment by Kadam (1936), and a separation ratio of 1:2:1 for homozygous hairiness: heterozygous hairiness: glabrous was observed in the F_3_ population, which concluded that glume hairiness gene is a dominant gene. Most studies indicated that glume pubescence was controlled by a single dominant allele in wheat (Sears, 1954; Tsunewaki and Jenkins, 1961; McIntosh and Bennett, 1978). However, the heavy pubescence in the Italian variety Loro was reported as an incompletely dominant allele in the study of Anderson and Mcginnis (1960), and the glume pubescence in durum wheat cv. Kahla was controlled by a recessive allele (Sheybani and Jenkins, 1961). The location analysis of *Hg1* (formerly named *Hg*) could date back to the 1960s, and the aneuploids of common wheat first identified the location of *Hg1* on chromosome 1A (Sears, 1954). Later, Tsunewaki (1962, 1966) confirmed this by the monosomic analysis; then, McIntosh and Bennett (1978) assigned *Hg1* to the short arm of chromosome 1A using telocentric mapping. *Hg1* was further located on a linkage map of chromosome 1AS in *T*. *monococcum* (Dubcovsky and Dvorak, 1995) and *T. aestivum* (Spielmeyer and Richards, 2004; Khlestkina et al., 2006).

With the development of molecular markers, such as simple sequence repeat (SSR), diversity array technology (DArT), single-nucleotide polymorphism (SNP), and the sequencing technology, the localization of *Hg1* in chromosome 1AS was more accurate and efficient using different methods, such as linkage mapping (Luo et al., 2016), transcriptome analysis (Luo et al., 2020), and genome-wide association studies (GWAS) (Sheoran et al., 2019; Wang et al., 2019). Luo et al. (2016) mapped *Hg1* in Tibetan semi-wild wheat (*T. aestivum* subsp. *tibetanum* Shao) accession Q1028 with SSR markers to a 3.3 cM region [physical region about 5 Mbp in IWGSC RefSeq v1.1 (Iwgsc et al., 2018), *Xsaufc2* (1A:1.37 Mbp)- *Xgwm136* (1A:6.42 Mbp)] on chromosome 1AS and further analyzed the candidate genes through a transcriptome analysis for glume hairiness in two sets of near-isogenic lines (NILs) of wheat (Luo et al., 2020). Wang et al. (2019) detected a SNP marker *IWA4754* [at chr1A: 12,369,432 bp in IWGSC RefSeq v1.1 (Iwgsc et al., 2018) and chr1A: 13,808,758 bp in IWGSC RefSeq v2.1 (Zhu et al., 2021)] that is significantly associated with glume pubescence (Gp). Although these studies provided useful and accurate information for fine mapping of *Hg1* and the identification of candidate genes in the wheat genome, the cloning of this gene has not yet been reported for the large complex wheat genome.

The recent release of high-quality genome (Iwgsc et al., 2018) and pan-genome data of wheat (Walkowiak et al., 2020), as well as the high-throughput genotyping projects (He et al., 2019; Guo et al., 2020; Hao et al., 2020; Zhou et al., 2020), provides the basis for a species-wide understanding of genome variations, which also facilitates the cloning of agriculturally important genes. With the fast development of the high-throughput genotyping platform and the substantial reductions in the price of sequencing, it is more approachable and efficient to perform gene mining and function analysis using GWAS and haplotype analysis combined with bioinformatics analysis in different studies (Yano et al., 2019; Abrouk et al., 2021; Hu and Zuo, 2021; Miculan et al., 2021; Tang et al., 2021). Here, we performed a GWAS between SNP makers and glume pubescence (GP) in 352 wheat accessions and further demonstrate the combination of the gene expression and haplotype analyses for isolating the *Hg1* gene on chromosome 1AS.

## Materials and Methods

### Materials

A set of 352 hexaploid wheat (*T. aestivum*) accessions with records in the Germplasm Resources Information Network (GRIN) database^1^ were selected from 1,026 diverse accessions of hexaploid and tetraploid wheat in the study of He et al. (2019). In the previous study, the 1,026 diverse accessions were sequenced using exome-sequencing technology to identify wild-relative introgression, selection for improvement and environmental adaptation, and mining alleles of agronomic genes explaining a substantial proportion of phenotypic variation. The selected 352 wheat accessions comprised of uncertain collections (58), wild (W:3), landraces (L:130), cultivars (C:75), genetic stocks (G:1), and improved breeding (B:85). Among them, 333, 12, 4, 2, and 1 accessions were of *T. aestivum*, *T. spelta*, *T. macha*, *T. sphaerococcum*, and *T. compactum*, respectively. The information of 352 selected hexaploid wheat accessions is listed in Supplementary Table 1.

### Phenotyping

Glume pubescence (Gp) is an important morphological trait with high heritability to distinguish/characterize wheat. The phenotype of Gp for the 352 wheat accessions was searched and downloaded from the GRIN database^2^ according to the accession IDs. The phenotype record of Gp was defined with a score of 1–9 (1 = ABSENT, 9 = LONG) according to the type and extent of glume pubescence in the website https://npgsweb.ars-rin.gov/gringlobal/descriptordetail?id=65010. The 352 accessions in this study were all recorded with four types (1 = ABSENT, 3 = EDGE ONLY, 5 = SHORT (FINE), 9 = LONG, READILY VISIBLE, Supplementary Figure 1) of Gp in the GRIN database. Moreover, the spike figures of the accessions in the website, which are clear enough to easily detect the glume with hairiness or not, were used to check and correct the glume hairiness phenotype of those with obvious wrong records. The details are shown in Supplementary Table 1.

### Single-Nucleotide Polymorphism Genotyping and Filtering

The SNP data were initially genotyped by He et al. (2019) in the 1000 wheat exome project using exome-sequencing technology, and the details about DNA isolation, exome capturing and sequencing, SNP calling, and filtering were provided. The reference genome used in their study is IWGSC RefSeq v1.1 (Iwgsc et al., 2018). We downloaded the VCF file (before imputation) from the website of the 1000 wheat exomes project^3^ and selected the genotype of 352 samples using the “bcftools view” function of BCFtools 1.8 software (Danecek et al., 2021). First, the SNPs with missing data >80% and MAF <1% were filtered by VCFtools 0.1.16 (Danecek et al., 2011). Second, the SNP data were imputed by Beagle (version: 21Apr21.304) (Browning et al., 2018) with the default parameters. Finally, a total of 2,368,251 SNPs with missing data <20% and MAF >0.05 were kept for further study by VCFtools 0.1.16 (Danecek et al., 2011).

### Population Genomic Analyses

Principal component analysis was performed using the glPca function of the package adegenet 2.1.5 (Jombart, 2008) in R version 4.0.1 (R Core Team, 2013)^4^. Structure analyses were performed with Structure 2.3.4 software (Hubisz et al., 2009) using a subset of 17,325 SNPs. This subset was selected by applying the following criteria: SNPs with linkage disequilibrium (LD) above 0.02 were removed using Plink “–indep-pairwise 1000 10 0.02.” A total of 50,000 burn-in periods followed by 100,000 Markov Chain Monte Carlo (MCMC) iterations from *K* = 1–10 clusters were used to identify the optimal cluster (*K*). Five independent runs were generated for each *K*. The results of the analysis were used as input to the Structure Harvester tool (Earl and Vonholdt, 2012) to predict the best *K*-value based on the Evanno method (Evanno et al., 2005). PHYLIP v3.5 (Felsenstein, 1993) was used to transfer the 17,325 SNPs data for generating the multiple sequence alignment file in PHYLIP format, and a phylogenetic tree was constructed using IQ-TREE (Nguyen et al., 2015) *via* a maximum-likelihood method with 1000 bootstrap replications. FigTree 1.4.4^5^ was used to optimize the visualization of the phylogenetic tree.

### Genome-Wide Association Study of Glume Pubescence

Genome-wide association studies for glume pubescence was conducted by the GAPIT package (Wang and Zhang, 2021) in R version 4.0.1 (R Core Team, 2013) (see text footnote 4) using the general linear model (GLM) (Price et al., 2006), the mixed linear model (MLM) (Zhang et al., 2005; Yu et al., 2006), the compressed MLM (CMLM) (Zhang et al., 2010), and the multiple loci mixed model (MLMM) (Segura et al., 2012). The Kinship (K) and PCA (P) for the methods were calculated using the GAPIT package. The first three principal components (PCs) were included in the GWAS model to correct for the hidden population structure. The threshold for *p*-value (*P* < 4.22 × 10^–9^) was corrected using the Bonferroni correction method (0.01 divided by the number of SNPs) (Li et al., 2012), following the study of Zhang et al. (2018). The significant associations, repeatedly detected in at least two methods, are viewed as reliable. If the associated SNPs revealed a single peak, they will be treated as a common QTN cluster (QTNc). According to the *p*-value and the effect of the associated SNP, the SNP with the lowest *p*-value and highest effect represents the peak SNP of the detected QTNc. The QTNc was named as “qtnc” + trait name abbreviation + chromosome + detected QTNc order on chromosome. Besides, the Manhattan plot was used for the visualization of association results by the CMplot package (Yin et al., 2021) in R 4.0.1 (R Core Team, 2013) (see text footnote 4).

### Single-Nucleotide Polymorphism Annotation

The genotype of significantly associated SNPs in the candidate gene regions for 352 wheat accessions was extracted from the initial genotype file of GWAS. The genome sequences and annotation file of IWGSC RefSeq v1.1 (Iwgsc et al., 2018) and IWGSC RefSeq v2.1 (Zhu et al., 2021) were downloaded from Wheat@URGI databases^6^ (Alaux et al., 2018) and used to annotate the SNP *via* the SnpEff v4 software (Cingolani et al., 2012). Those genes in which SNPs were annotated with loss-of-function mutations described in the study of Torkamaneh et al. (2018) or were located in 5′ UTR, 3′ UTR, and promotor regions will be considered as reliable candidate genes.

### Putative Candidate Gene Analysis and Expression Data

To find the candidate gene of *Hg1*, the associated region of the detected QTNc on chromosome 1AS was considered as the candidate gene region for *Hg1*. The candidate genes were selected according to the functional annotation (IWGSC RefSeq 1.1) of the genes in the candidate region (Iwgsc et al., 2018), and the transcriptome datasets of IWGSC (2014)I and Li et al. (2018) downloaded from the website of WheatOmics 1.0^7^ (Ma et al., 2021) were used to select the candidate genes with high expression in spikelet and glume.

### Haplotype Analysis of *TaELD1-1A* in Wheat Population

To assess the allelic variation of the *TaELD1-1A* gene across various wheat cultivars, the haplotype analysis of *TaELD1-1A* was performed using the SNP data (heterozygosity <0.03) on *TaELD1-1A* gene sequences among the 352 wheat accessions retrieved from the 1000 wheat exomes project of He et al. (2019) (see text footnote 3) using the “CandiHap” package (Li et al., 2020) of R 4.0.1 (R Core Team, 2013) (see text footnote 4), and the differences of the phenotypes for Gp corresponding to different haplotypes were tested. Moreover, the homologous gene sequences of *TaELD1-1A* in pan-genomes including 10+ hexaploid wheat (Walkowiak et al., 2020), emmer wheat (Zavitan) (Avni et al., 2017), and durum wheat (Svevo) (Maccaferri et al., 2019) genomes were downloaded from Ensembl Plants^8^ according to the best-match gene IDs to *TraesCSU02G143200* through BLAST. The above SNPs of *TaELD1-1A* among the pan-genomes were obtained by alignment and were used to analyze the haplotypes of *TaELD1-1A* among pan-genome accessions.

## Results

### The Phenotypic Variation of Glume Pubescence

The phenotype of Gp for the 352 wheat accessions was obtained from the Germplasm Resources Information Network (GRIN) database (see text footnote 1) according to the accession IDs (Supplementary Table 1). The spike figures of the accessions in the GRIN database, which are clear enough to easily detect the glume with hairiness or not, were used to check and correct the ones with obvious wrong records. GP was scored on a range of 1–9 (1 = ABSENT and 9 = LONG) according to the type and extent of glume pubescence in the GRIN database^9^, where only four types (1 = ABSENT, 3 = EDGE ONLY, 5 = SHORT (FINE), 9 = LONG, READILY VISIBLE) (Supplementary Figure 1) of Gp were recorded among 352 accessions in this study. Among 352 wheat accessions, 315, 11, 11, and 15 accessions belonged to type 1, 3, 5, and 9 Gp, respectively. The percentage of type 1 (1 = ABSENT) and type 2 (3 = EDGE ONLY) Gp were increased from landrace to cultivar, while the percentage of type 3 [5 = SHORT (FINE)] and type 4 (9 = LONG, READILY VISIBLE) were decreased (Table 1). This suggested that Gp has been under selection during the improvement from landrace to cultivar on some extent.

**TABLE 1:** Distribution of different types of Gp among wild, landrace, and cultivar wheat.

Gp	Cul/(%)	Landrace/(%)	Wild/(%)	Uncertain/(%)	Sum/(%)
1	149 (92.5)	113 (86.9)	3 (100)	50 (86.2)	315 (89.5)
3	7 (4.35)	2 (1.5)	0	2 (3.4)	11 (3.1)
5	3 (1.86)	5 (3.9)	0	3 (5.2)	11 (3.1)
9	2 (1.24)	10 (7.7)	0	3 (5.2)	15 (4.3)
Sum	161	130	3	58	352

### Genotypic Features and Population Structure

**FIGURE 1:**
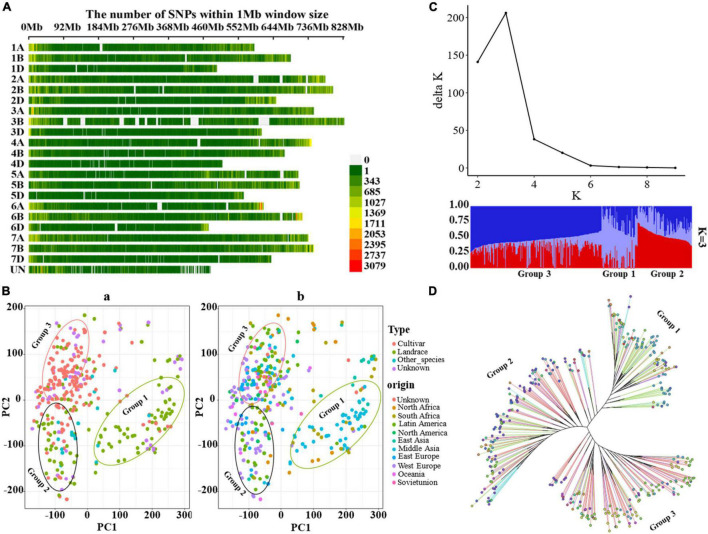
Genotypic features and population structure of bread wheat. **(A)** The distribution of SNPs across the entire genome, the color legend indicates the SNP number. **(B)** Principal component analysis (PCA) across the 352 wheat accessions, the color legend “Type” is for [**(B)**a], “Other_species” is for the relatives (19 accessions) of *T. aestivum*, “Unknown” is for missing record for cultivar or landrace, the color legend “origin” is for [**(B)**b], “Unknown” is for missing record of origin. **(C)** Population structure for 352 accessions, the optimal cluster (K) was *K* = 3. **(D)** Maximum-likelihood tree constructed with IQ-tree, **(D)** shared the same color legend with **(B)**, and the color legends “Type” and “origin” refer to the branches and the nodes at the end of the branches in the tree, respectively.

After filtering, a total of 2,368,251 SNPs covering the whole genome were obtained for 352 wheat populations. The distribution of SNPs on different chromosomes was visualized by the CMplot package (Yin et al., 2021) in R version 4.0.1 (R Core Team, 2013) (see text footnote 4) (Figure 1A). The SNPs were distributed across the entire genome, with increased frequency in gene-rich, telomeric regions (Figure 1A). Of the 2,368,251 variants, 863,242, 1,083,108, and 356,899 were located on the A, B, and D subgenomes, respectively, whereas 65,002 variants were unanchored (chrUn), and the number of SNPs on chromosomes varies from 18,652 on chr4D to 198,349 on chr2B; the average SNP density (numbers of SNPs per Mbp) ranges from 36.58 (chr4D) to 247.55 (chr2B) (Supplementary Table 2). Principal component analysis (PCA) revealed a separation of 352 wheat accessions into three gene pools comprising accessions as old landraces, a mixture of landraces and cultivar, and modern cultivars (Figure 1B). The phylogeny and structure analyses showed similar results (Figures 1C,D). The first principal component mainly separated the landraces of Middle Asia from the landraces and cultivars of other places, and the second axis mainly separated the wheat accessions into two pools: the landraces of Europe and Latin America, and the modern cultivars. Similarly, a maximum-likelihood tree also provided evidence for three groups: group 1 included mainly landraces from Middle Asia, group 2 consisted of most landraces and a few cultivars mainly from Europe and Latin America, and group 3 were mostly cultivars from different places. In fact, some accessions showed discrepancies between their indicated accession type (or geographical origin) and the PCA cluster. The likely reasons for this are erroneous passport information or mistakes during the dissemination of the GenBank materials. Alternatively, this pattern might reflect the interchange of germplasm between different regions before collection. Moreover, the optimal cluster (K) for population structure was defined as *K* = 3 (Figure 1C). The PCA, phylogenetic tree, and structure population showed similar results, revealing three gene pools for the wheat populations. The accession type and geographical origin confirmed that the bread wheat originated from Middle Asia, which was then domesticated and spread to Europe, Asia, United States, and Africa (Pont et al., 2019; Zhou et al., 2020).

### Genome-Wide Association Studies to Identify Single-Nucleotide Polymorphisms Associated with Glume Pubescence

**FIGURE 2:**
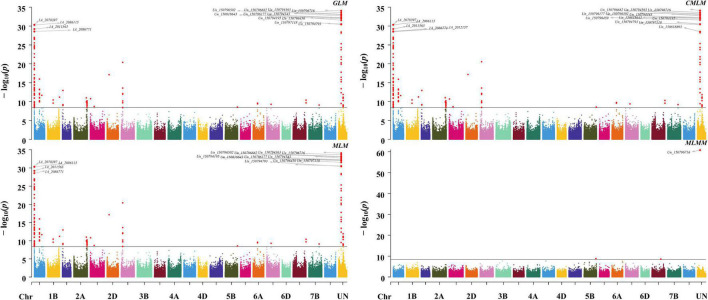
Manhattan plots of GWAS for Gp using GLM, MLM, CMLM, and MLMM methods. The associated SNPs with *p*-value ≤1 × 10^– 28^ were tagged on the plots.

To detect the most significant marker–trait associations, four models including three single loci methods (GLM, MLM, and CMLM) and one multiple loci method (MLMM) were employed to conduct the GWAS. A total of 148 significant associations were co-detected among three single loci methods, among which eight QTN clusters (QTNcs) for Gp were detected on chromosomes 1A, 1B, 1D, 2A, 3A, 6A, 7A, and Un (Figure 2 and Supplementary Table 3) (*p*-value = 0.01/number of SNPs = 4.22 × 10^–9^). It is worth noting that two obvious single peaks on 1A and Un were co-detected by GLM, MLM, and CMLM. These two peaks were located in the confidence intervals of ∼0.85 Mb (spanning physical positions 1A: 1.24–2.09 Mb) and ∼0.027 Mb (Un: 150.79–150.82 Mb) in the IWGSC RefSeq v1.1, respectively. Through BLAST, the collinear positions for the two peaks (1A: 1.24–2.09 Mb and Un: 150.79–150.82 Mb) were all located on the short arm of chromosome 1A in IWGSC RefSeq v2.1 and WEWSeq_v.1.0 referring to one peak with an interval of ∼1.77 Mb (1A: 1.23–3.00 Mb IWGSC RefSeq v2.1) (Supplementary Table 3). Therefore, these two association peaks should be one QTNc (named *qtnc_Gp_1A1*) for Gp on chromosomes 1AS, which explained 9.9–51.3% of phenotypic variation (Supplementary Table 3). This physical position coincides with the *Hg1* locus of previous reports (Luo et al., 2016, 2020). Moreover, the peak SNP *Un_150796716*, explaining the highest (51.3%) phenotypic variation, was co-detected among the four model methods, suggesting that a more reliable candidate gene for the *Hg1* gene might be near this SNP.

### Candidate Gene Analysis for *Hg1*

According to the genome annotations of IWGSC RefSeq v1.1 and IWGSC RefSeq v2.1, we compared the genes in the associated region 1A: 1.24–2.09 Mb and Un: 150.79–150.82 Mb of IWGSC RefSeq v1.1 with that in the collinear region 1A:1.23–3.00 Mb of IWGSC RefSeq v2.1. A total of 58 genes, including 20 high and 38 low confidence genes, were detected in the target region of *Hg1* in IWGSC RefSeq v1.1, among which 18 genes were not anchored to the target region of IWGSC RefSeq v1.1 but belong to the region (1A:1.23–3.00 Mb) of IWGSC RefSeq v2.1. These genes may be incorrectly assembled to the wrong positions in IWGSC RefSeq v1.1 and were corrected in IWGSC RefSeq v2.1 (Supplementary Table 4). Moreover, four genes *TraesCSU02G231400LC*, *TraesCSU02G231300LC*, *TraesCSU02G231200LC*, and *TraesCSU02G143200* were not annotated in IWGSC RefSeq v2.1 (Supplementary Table 4).

**FIGURE 3:**
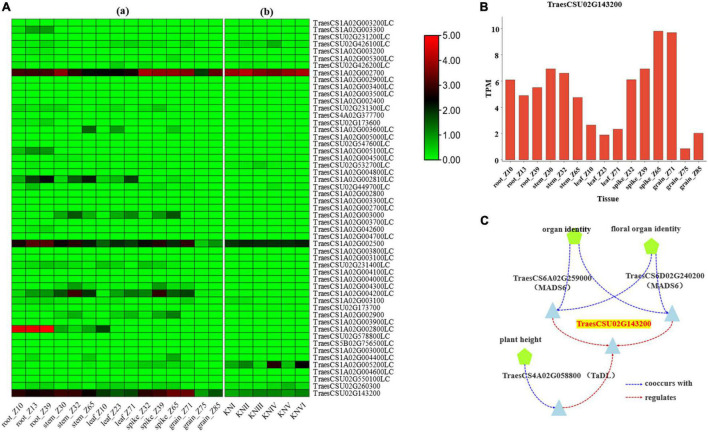
Expression data for candidate genes. **(A)** The expression pattern of the candidate genes in different tissues and developmental stages: [**(A)**a] refers to the expression data from IWGSC (2014), [**(A)**b] refers to the expression data from Li et al. (2018), and KNI-KNVI represent the spikes in different developmental stages. **(B)** The expression of *TraesCSU02G143200* gene in different tissues of wheat from the data of IWGSC (2014). **(C)** The co-expression data of *TraesCSU02G143200* from knetMiner (https://knetminer.com/Triticum_aestivum/).

The expression pattern of the genes in different tissues and developing spike period were obtained from the research of IWGSC (2014) and Li et al. (2018) through website tools (see text footnote 7). The results identified six genes, namely *TraesCSU02G426100LC*, *TraesCS1A02G002700*, *Tra esCS1A02G003000*, *TraesCS1A02G002500*, *TraesCS1A02G0052 00LC*, and *TraesCSU02G143200* with high expression in spike (Figure 3A).

Function annotation for associated SNPs in the region of *Hg1* was conducted by SnpEff software with IWGSC RefSeq v1.1 and IWGSC RefSeq v2.1 annotation files, and the result showed that sixteen associated SNPs on seven genes (*TraesCS1A02G002400, TraesCS1A02G002500*, *TraesCS1A02G002700, TraesCSU02G143200*, *TraesCSU02G2314 00LC*, *TraesCSU02G173700*, and *TraesCS1A02G005300LC*) were with function variations (Supplementary Table 3). According to the annotation of associated *SNPs* and the expression pattern of the genes, *TraesCS1A02G002500*, *TraesCS1A02G002700*, and *TraesCSU02G143200* may be the candidate genes for *Hg1*. According to the gene function annotation, *TraesCSU02G143200* (*TaELD1-1A*, encoding glycosyltransferase-like ELD1/KOBITO 1) (Supplementary Table 4), involved in the regulation of cell elongation (Cheng et al., 2000; Pagant et al., 2002), was identified as the most promising candidate gene for *Hg1*. Two associated SNPs (*Un_150794343* and *Un_150797000*), with function variations present in *TraesCSU02G143200*, may result in its function changes. *TraesCSU02G143200* showed a relatively high expression in spike/spikelet (Figures 3A,B). The co-expression data of *TraesCSU02G143200* from knetMiner^10^ showed that it was co-expressed with *MADS6* (*TraesCS6A02G259000* and *TraesCS6D02G240200*) and *TaDL* (*TraesCS4A02G058800*) in floral organ (spike) and involved in the regulation of the development of floral organ under the regulation of *MADS6* and *TaDL* (Figure 3C). Moreover, glycosyltransferase-like protein ELD1/KOB1 has been identified to play an important role in the regulation of cell elongation, affecting the development of root hairs, and the root hair density of the mutants was significantly greater than that of the wild type in *Arabidopsis* (Cheng et al., 2000; Pagant et al., 2002). Therefore, *TraesCSU02G143200* (*TaELD1-1A*) is the most promising candidate gene of *Hg1* that may be involved in the regulation of glume pubescence.

### Haplotype Analysis of *TaELD1-1A*

**FIGURE 4:**
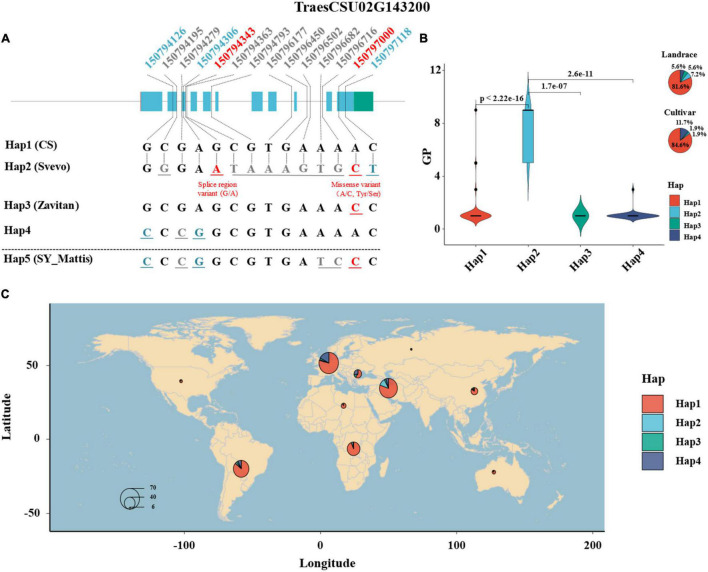
Haplotype analysis of *TraesCSU02G143200* in 352 wheat accessions and the pan-genomes. **(A)** The haplotype and sequence analysis of *TraesCSU02G143200* among 352 wheat accessions, involving 10+ hexaploid wheat and the tetraploid wheat Zavitan and Svevo reference genomes. The numbers at the top indicate the genomic positions of the SNPs on *TraesCSU02G143200* among the population; the numbers and the corresponding base letters with gray color indicate that the SNP variants are in the introns of *TraesCSU02G143200*; the numbers and corresponding base letters with blue or red color indicate that the SNP variants are in the exons of *TraesCSU02G143200*, blue means the SNPs with synonymous mutations, red means the two SNPs with function mutations on *TraesCSU02G143200*, a splice region variant (G/A) at Un:150794343 and a missense variant (A/C, Tyr/Ser) at Un:150797000. **(B)** The haplotypes of *TraesCSU02G143200* and theirs Gp score comparison and frequency distribution among 352 wheat accessions. **(C)** The geographical distribution and frequency of the haplotypes of *TraesCSU02G143200*. The size of the pie is proportional to the sample size.

A total of 52 SNPs on the gene sequence of *TaELD1-1A* among the 352 wheat accessions were retrieved from the 1000 wheat exomes project of He et al. (2019) (Supplementary Table 5), among which 14 SNPs were filtered with heterozygosity <0.03 and used for haplotype analysis by the “CandiHap” package (Li et al., 2020) of R 4.0.1 (R Core Team, 2013) (see text footnote 4) (Supplementary Table 6). Haplotype analysis showed that four main haplotypes (Hap1–4, containing accessions >10) of *TaELD1-1A* were detected among 352 wheat accessions (Figure 4 and Supplementary Table 7). Moreover, according to the 14 SNPs information, four haplotypes (Hap1, Hap2, Hap3, and Hap5) for *TaELD1-1A* were detected among 10+ pan-genomes, durum wheat (Svevo), and emmer wheat (Zavitan), and their phenotypes of glume pubescence were consistent with that in the 352 wheat accessions (Figure 4 and Supplementary Table 8). The Hap1 is the haplotype of reference *TaELD1-1A* without variation, which was presented in CS, Cadenza, CDC Stanley, CDC Landmark, Claire, Jagger, Julius, Lancer, Mace, Norin61, Paragon, Spelt (PI 190962), and Weebill 1 and identified to be with type 1 (1 = ABSENT) glume pubescence (Supplementary Table 8). The Claire, Cadenza, and Paragon were the varieties of United Kingdom that were reported to have the glume with a smooth external surface^11^, and the CDC Stanley and CDC Landmark were the varieties of Canada that were recorded with glabrous glume and glabrous to very slightly pubescent glume^12^. The Hap2 is the haplotype of *TaELD1-1A* with function variants at Un:150794343 (splice region variant: G/A) and Un:150797000 (missense variant: A/C, Tyr/Ser), which include the type 3 [5 = SHORT (FINE)] and type 4 (9 = LONG, READILY VISIBLE) (Figures 4A,B and Supplementary Table 7). Svevo was a durum wheat belonging to Hap2, which has visible glume hairs (rough surface) (personal communication with Assaf Distelfeld and Elisabetta Mazzucotelli)^13^ ^,14^. Hap3 is the haplotype of *TaELD1-1A* with function variation Un:150797000 (missense variant: A/C, Tyr/Ser), which is presented in Zavitan (personal communication with Assaf Distelfeld and Elisabetta Mazzucotelli) and Robigus^15^ with the glume having a smooth external surface (type 1, 1 = ABSENT). Hap4 is the haplotype of *TaELD1-1A* with synonymous variants at Un:150794126 and Un:150794306, which is presented with type 1 (1 = ABSENT) glume pubescence. Hap5 is a combination of Hap3 and Hap4 missing among 352 accessions, which is presented in SY Mattis and ArinaLrFor (personal communication with Simon Krattinger and Lamia Aouini) with type 1 (1 = ABSENT) glume pubescence. The haplotype analysis suggests that the function variation at Un:150794343 (splice region variant: G/A) on *TaELD1-1A* may be the key variation that affects the transcript of *TaELD1-1A* and then affects Gp. Therefore, the haplotype analysis of *TaELD1-1A* in 352 wheat accessions and the pan-genomes further indicated *TraesCSU02G143200* (*TaELD1-1A*) as the most promising candidate gene of *Hg1*. Moreover, among 352 accessions, the frequency of Hap1 and Hap4 increased from landrace (81.6 and 5.6%) to cultivar (84.6 and 11.7%), while the frequency of Hap2 and Hap3 decreased from landrace (7.2 and 5.6%) to cultivar (1.9 and 1.9%), suggesting that *TaELD1-1A* was under selection according to the visible phenotype marker (Gp) during the improvement from landrace to cultivar on some extent (Figure 4B). Some individual discrete points in the haplotypes (such as Hap1) may be incorrectly recorded in the database, although we have revised some incorrect records of the Gp according to the spike images of the accessions in the GRIN database (Figure 4B and Supplementary Table 1). The geographical distribution of the haplotypes of *TraesCSU02G143200* showed that Hap2 accessions with visible glume hairs were mainly from Middle Asia (Figure 4C).

## Discussion

### *TaELD1-1A*, the Most Reliable Candidate Gene for *Hg1*

Because of its striking phenotype and importance for distinguishing/characterizing wheat, the genetic inheritance of glume hairiness or pubescence and the localization of *Hg1* were systematically studied after the rediscovery of Mendel’s laws in the early 1900s (Biffen, 1905). With the development of molecular markers and sequencing technology, several studies (Luo et al., 2016, 2020; Sheoran et al., 2019) have provided more accurate and efficient information for fine mapping of *Hg1* on chromosome 1AS in wheat in the recent years. However, the cloning for *Hg1* has not yet been reported to date. According to the fast development of the high-throughput genotyping platform and the substantial reduction in the price of sequencing, GWAS combined with bioinformatics analysis becomes a powerful and efficient tool for mining genetic loci associated with any trait, including quantitative and qualitative traits. In this study, we performed a GWAS between SNP makers and glume pubescence (Gp) in 352 wheat populations with exon sequencing and further demonstrated the gene expression and haplotype analyses for isolating the *Hg1* gene. First, two significantly associated peaks (1A: 1.24–2.09 Mb and Un: 150.79–150.82 Mb, IWGSC RefSeq v1.1) for Gp were detected, and the region of Un: 150.79–150.82 Mb was certificated to be in the region of 1A: 1.24–2.09 Mb as one QTNc (*qtnc_Gp_1A1*) for Gp through collinearity analysis with the wheat genome IWGSC RefSeq v2.1 and emmer wheat genome WEWSeq_v.1.0, which coincided with the *Hg1* locus of previous reports (Luo et al., 2016, 2020); second, according to the annotation, expression pattern, and function SNP variation of the candidate genes in the target region, *TraesCSU02G143200* (*TaELD1-1A*) encoding glycosyltransferase-like ELD1/KOBITO 1 was inferred as the most promising candidate gene of *Hg1* that may be involved in the regulation of glume pubescence; third, the haplotype analysis of *TraesCSU02G143200* among the GWAS population and its co-expression with *MADS6* and *TaDL* in the regulation of floral organ (spike) development from knetMiner website (see text footnote 10) further inferred that *TraesCSU02G143200* was the candidate gene of *Hg1*. Moreover, glycosyltransferase−like protein ELD1/KOB1 of *Arabidopsis* has been identified to play an important role in the regulation of cell elongation, affecting the development of root hairs, and the root hair density of the mutants was significantly greater than that of the wild type in *Arabidopsis* (Cheng et al., 2000; Pagant et al., 2002). Although multiple lines of evidence indicated that *TraesCSU02G143200* is a reliable candidate gene for the *Hg1*, it needs to be further verified through gene overexpression and knockout experiments.

### The Importance of High-Quality Reference Genome and Annotation Information for Gene Mining

Since *Hg1* was first located on the short arm of chromosome 1A in the 1960s (Sears, 1954; Tsunewaki, 1966), the fine mapping of *Hg1* in the telomere region of chromosome 1AS was more accurate and efficient using different methods, such as linkage mapping (Luo et al., 2016), transcriptome analysis (Luo et al., 2020), and genome-wide association studies (GWAS) (Sheoran et al., 2019). However, the gene for *Hg1* has not yet been cloned. We speculate that there may be several reasons that limit the fine mapping and cloning of the *Hg1* gene. First, many duplicate sequences exist in the region of *Hg1*, resulting in difficulty for the polymorphism makers exploring and fine mapping; second, the duplicate sequences resulted in miss- or un-assembled sequences for this region, among which *TraesCSU02G143200* (located at chrUn:150793569–150797591 of IWGSC RefSeq v1.1) was detected in the region of the *Hg1* locus according to the blast results to IWGSC RefSeq v2.1, WEWSeq_v.1.0 genomes, and pan-genomes of wheat (Supplementary Table 3 and Supplementary Figure 2); third, duplicate sequences affect the gene annotation in this region, although some assembly errors such as the contigs on chrUn in IWGSC RefSeq v1.1 were reassembled to the corresponding position in IWGSC RefSeq v2.1, while *TraesCSU02G143200* was missing (sequence without gene annotation) in IWGSC RefSeq v2.1 (Supplementary Table 4). Therefore, the high-quality reference genome and annotation information are very important for gene mining, and the un-contig sequences in chrUn should not be neglected.

### Possible Selection and Domestication Trend of Glume Pubescence

Glume hairiness or pubescence is an important morphological trait with high heritability and is frequently used as a morphological marker to distinguish/characterize wheat; therefore, *Hg1* may have been under selection according to the visible phenotype marker (Gp) during the domestication and improvement of wheat on some extent. The haplotype analysis of *TaELD1-1A* suggested that a weaker selection existed during the improvement from landrace to cultivar (Figure 4). The genetic diversity, differentiation, and selection parameters such as Fst, Π, Tajima’s D, and XP-CLR among landrace and cultivar wheat and wild and domesticated emmer in some studies (Avni et al., 2017; He et al., 2019) indicated that the region of *Hg1* was identified with high genetic diversity, low differentiation, and weak or no selection pressure. This indicated that the region of *Hg1* might not been undergone selection. The possible explanations may be as follows: many genes/loci (including genes that are beneficial or unfavorable to production), such as barley yellow dwarf virus (BYDV) resistant gene (Wu et al., 1999), powdery mildew resistance gene (*Pm3*) (Briggle and Sears, 1966), leaf rust locus (Howes, 1986) and Karnal bunt locus (Warham, 1988), tiller inhibition gene (*Tin*) (Richards, 1988; Spielmeyer and Richards, 2004), *Gli-A1* locus (Howes, 1986), and abiotic stress gene loci to cold and drought (Trethowan et al., 1998; Pshenichnikova et al., 2019) were gathered in or beside the region of *Hg1* and shown to be linked to *Hg1*, and they have been positively or negatively selected during wheat breeding, resulting in counteracting selection pressure in the region of *Hg1*. For example, hairy glume can be used as a morphological marker for powdery mildew resistance (*Pm3*) because of its tight linkage with *Pm3* (Briggle and Sears, 1966), which leads to the positive selection for *Hg1*. Meanwhile, the hairy glume phenotype can also be used as a marker for the low tillering gene (*Tin*) (Richards, 1988; Spielmeyer and Richards, 2004), which leads to the negative selection for *Hg1*. Many important genes are clustered in this region resulting in a balance between the positive and negative selection in this region, so the selection pressure of this region was too weak to be detected. Therefore, hairy glume is not an obvious domestication trait like brittle rachis (*Br*), tough glume (*Tg*), and free-threshing (*Q*) during the evolution and domestication of wheat. Furthermore, there may be an imbalance in the selection of *Hg1* locus in different wheat populations of different origins, and a certain degree of selection signal may be detected.

## Conclusion

In this study, we performed a GWAS between SNP makers and glume pubescence (Gp) in a wheat population with 352 lines and further demonstrated the gene expression and haplotype analyses for isolating the *Hg1* gene. Eight QTNcs were detected significantly associated with Gp, among which one reliable QTNc (named *qtnc_Gp_1A1*) was detected referring to the *Hg1* locus, which can explain 9.9–51.3% phenotypic variation. According to the annotation, expression pattern, and function SNP variation of the candidate genes in the target region, *TraesCSU02G143200* (*TaELD1-1A*), encoding glycosyltransferase-like ELD1/KOBITO 1, was inferred as the most promising candidate gene of *Hg1* that may be involved in the regulation of glume pubescence. Moreover, haplotype analysis of *TraesCSU02G143200* among the GWAS population and pan-genome accessions and its co-expression with *MADS6* and *TaDL* in the regulation of floral organ (spike) development from knetMiner website (see text footnote 10) also support our prediction. Although multiple lines of evidence indicated that *TraesCSU02G143200* is a reliable candidate gene for the *Hg1*, it needs to be further verified through gene overexpression and knockout experiments. Moreover, our results revealed that many duplicate sequences exist in the region of *Hg1*, leading to the difficulty in fine mapping and cloning of *Hg1.* In addition, *TraesCSU02G143200* on chrUn was one of the un-assembled genes in chr1AS (Supplementary Figure 2 and Supplementary Table 4), suggesting that the information in chrUn is also very important and should not be neglected. Our study highlights the importance of high-quality reference genome and annotation information, as well as pan-genome information for gene cloning in wheat. Many duplicate sequences in the region of the *Hg1* locus were not well assembled which resulted in *TaELD1-1A* located chrUn in IWGSC RefSeq v1.1 and miss-annotated in IWGSC RefSeq v2.1. The accurate information and allelic variation at this locus would have remained hidden without access to the high-quality pan-genomes and relative genomes. As demonstrated in this study, the completion of these high-quality genomes and annotation information, as well as the bioinformatics analysis, represents a step change for gene cloning in wheat.

## Data Availability Statement

The original contributions presented in this study are included in the article/Supplementary Material, further inquiries can be directed to the corresponding author/s.

## Author Contributions

JZ: conceptualization and review and editing. XH: RNA-seq, SNP data acquisition and analysis, and writing original draft. XH and JZ: GWAS, haplotype analysis, and visualization. Both authors read and agreed to the published version of the manuscript.

## Conflict of Interest

The authors declare that the research was conducted in the absence of any commercial or financial relationships that could be construed as a potential conflict of interest.

## Publisher’s Note

All claims expressed in this article are solely those of the authors and do not necessarily represent those of their affiliated organizations, or those of the publisher, the editors and the reviewers. Any product that may be evaluated in this article, or claim that may be made by its manufacturer, is not guaranteed or endorsed by the publisher.

## Footnotes

1
https://npgsweb.ars-grin.gov/

2
https://npgsweb.ars-grin.gov/gringlobal/search

3
http://wheatgenomics.plantpath.ksu.edu/1000EC/

4
http://www.r-project.org

5
http://tree.bio.ed.ac.uk/software/figtree/

6
http://wheat-urgi.versailles.inra.fr/

7
http://wheatomics.sdau.edu.cn/expression/wheat.html

8
http://plants.ensembl.org/index.html

9
https://npgsweb.ars-grin.gov/gringlobal/descriptordetail?id=65010

10
https://knetminer.com/Triticum_aestivum/

11
https://wheat.agricrops.org/varieties/view

12
https://inspection.canada.ca

13
https://www.caione.it/prodotto/svevo/

14
https://www.sciencephoto.com/media/810982/view/ripe-svevo-durum-wheat-triticum-durum

15
https://wheat.agricrops.org/varieties/view/Robigus
