# Supplementary material for: Population Genomics and Haplotype Analysis in Bread Wheat Identify a Gene Regulating Glume Pubescence
Source: Front Plant Sci. 2022 Jul 13;13:897772. doi: 10.3389/fpls.2022.897772 (PMC9328021; doi:10.3389/fpls.2022.897772)
Supplement: Supplementary file 2 [file Presentation_1.PPTX]

## Slide 1
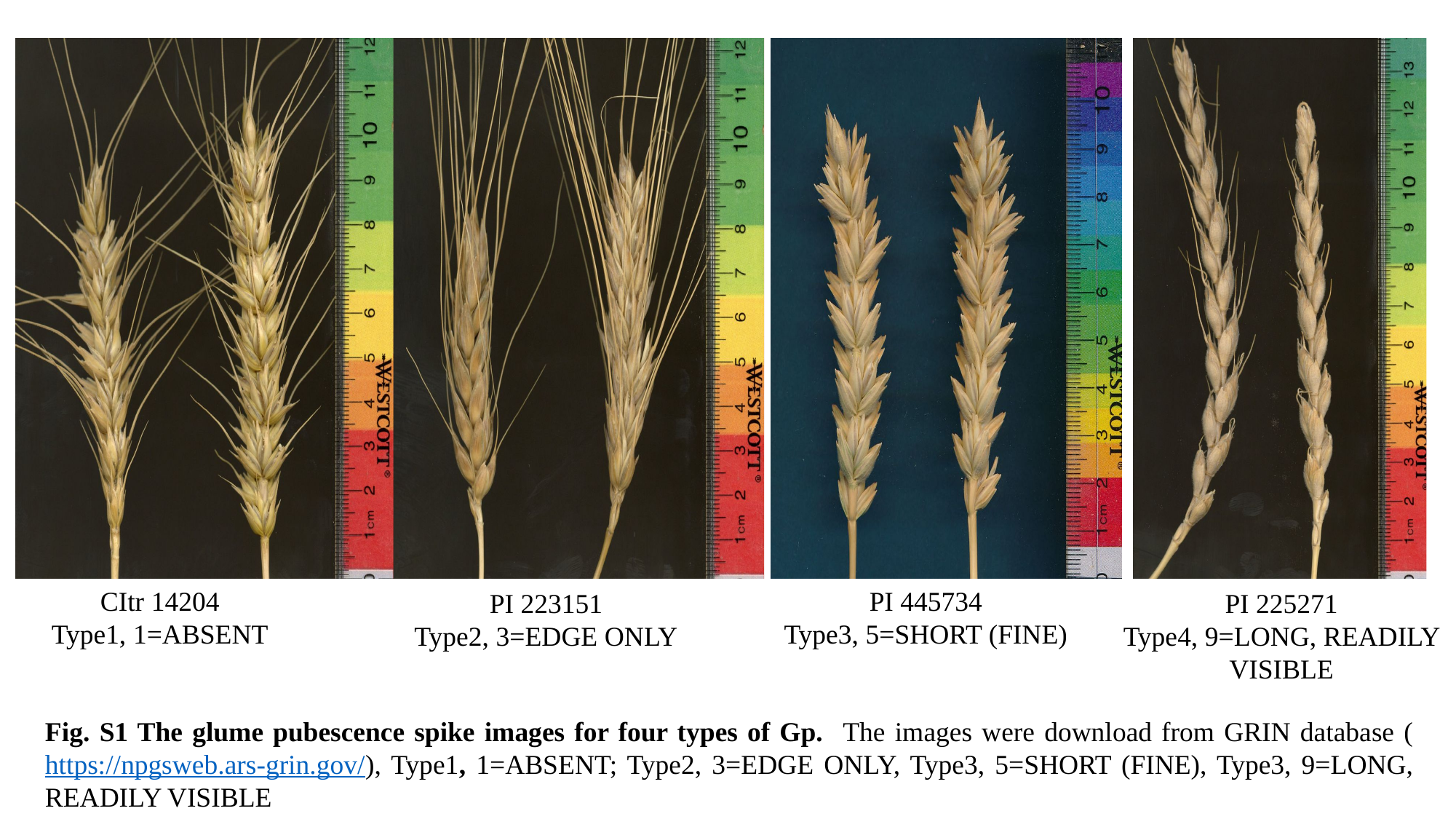

CItr 14204
Type1, 1=ABSENT
PI 445734
Type3, 5=SHORT (FINE)
PI 223151
Type2, 3=EDGE ONLY
PI 225271
Type4, 9=LONG, READILY VISIBLE
Fig. S1 The glume pubescence spike images for four types of Gp. The images were download from GRIN database (https://npgsweb.ars-grin.gov/), Type1, 1=ABSENT; Type2, 3=EDGE ONLY, Type3, 5=SHORT (FINE), Type3, 9=LONG, READILY VISIBLE
