# Supplementary material for: Population Genomics and Haplotype Analysis in Bread Wheat Identify a Gene Regulating Glume Pubescence
Source: Front Plant Sci. 2022 Jul 13;13:897772. doi: 10.3389/fpls.2022.897772 (PMC9328021; doi:10.3389/fpls.2022.897772)
Supplement: Supplementary file 3 [file Presentation_2.PPTX]

## Slide 1
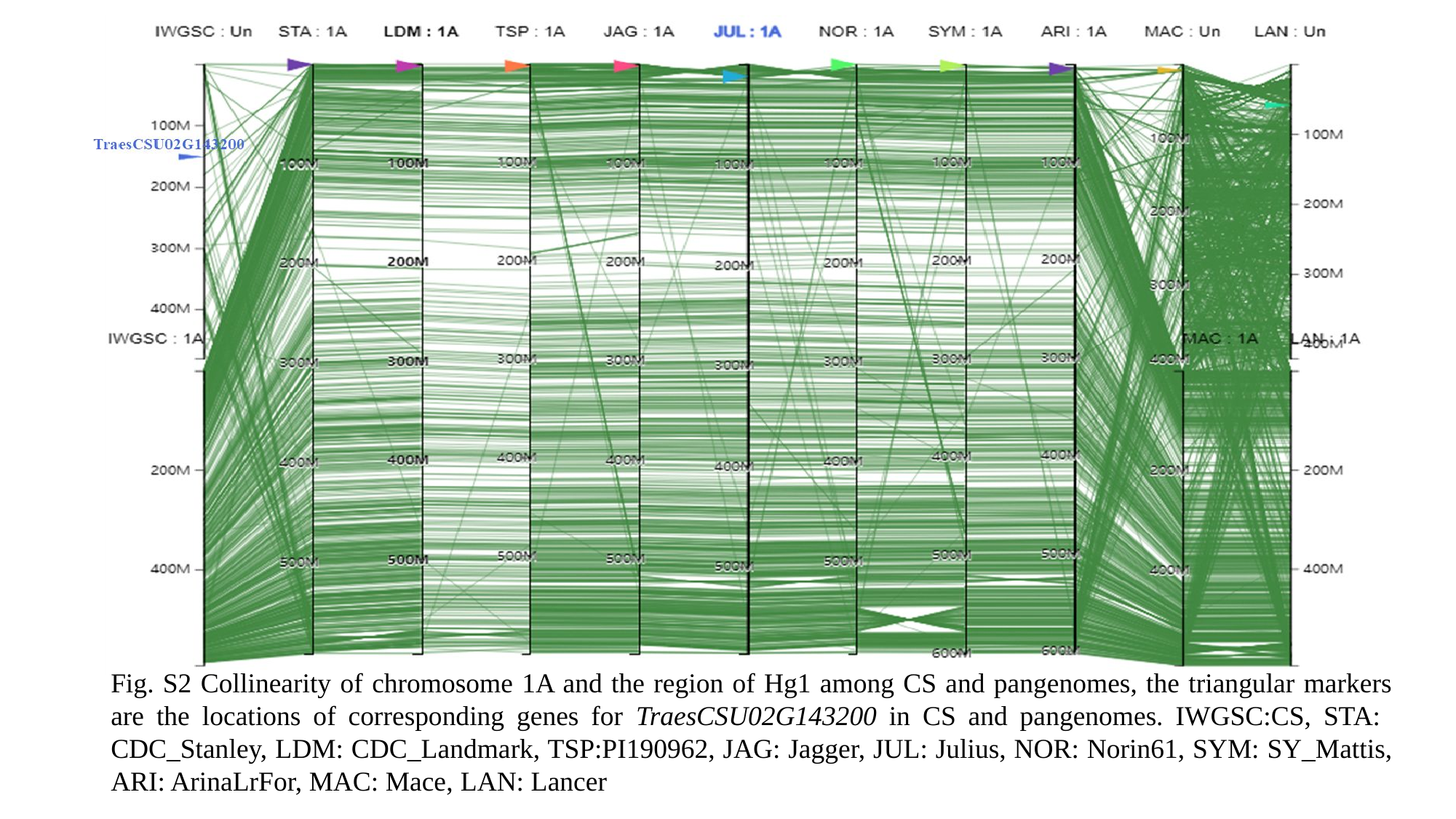

Fig. S2 Collinearity of chromosome 1A and the region of Hg1 among CS and pangenomes, the triangular markers are the locations of corresponding genes for TraesCSU02G143200 in CS and pangenomes. IWGSC:CS, STA: CDC_Stanley, LDM: CDC_Landmark, TSP:PI190962, JAG: Jagger, JUL: Julius, NOR: Norin61, SYM: SY_Mattis, ARI: ArinaLrFor, MAC: Mace, LAN: Lancer
